# Evaluation of Iodine-123 and Iodine-131 SPECT activity quantification: a Monte Carlo study

**DOI:** 10.1186/s40658-021-00407-1

**Published:** 2021-08-19

**Authors:** Michaella Morphis, Johan A. van Staden, Hanlie du Raan, Michael Ljungberg

**Affiliations:** 1 Department of Medical Physics, Faculty of Health Sciences, University of the Free State, PO Box 339, Bloemfontein, 9300 South Africa; 2 Medical Radiation Physics, Lund University, Lund, Sweden

**Keywords:** Monte Carlo simulations, SIMIND, Quantification accuracy, ^123^I, ^131^I, SPECT

## Abstract

**Purpose:**

The quantitative accuracy of Nuclear Medicine images, acquired for both planar and SPECT studies, is influenced by the isotope-collimator combination as well as image corrections incorporated in the iterative reconstruction process. These factors can be investigated and optimised using Monte Carlo simulations. This study aimed to evaluate SPECT quantification accuracy for ^123^I with both the low-energy high resolution (LEHR) and medium-energy (ME) collimators and ^131^I with the high-energy (HE) collimator.

**Methods:**

Simulated SPECT projection images were reconstructed using the OS-EM iterative algorithm, which was optimised for the number of updates, with appropriate corrections for scatter, attenuation and collimator detector response (CDR), including septal scatter and penetration compensation. An appropriate calibration factor (CF) was determined from four different source geometries (activity-filled: water-filled cylindrical phantom, sphere in water-filled (cold) cylindrical phantom, sphere in air and point-like source), investigated with different volume of interest (VOI) diameters. Recovery curves were constructed from recovery coefficients to correct for partial volume effects (PVEs). The quantitative method was evaluated for spheres in voxel-based digital cylindrical and patient phantoms.

**Results:**

The optimal number of OS-EM updates was 60 for all isotope-collimator combinations. The CF_point_ with a VOI diameter equal to the physical size plus a 3.0-cm margin was selected, for all isotope-collimator geometries. The spheres’ quantification errors in the voxel-based digital cylindrical and patient phantoms were less than 3.2% and 5.4%, respectively, for all isotope-collimator combinations.

**Conclusion:**

The study showed that quantification errors of less than 6.0% could be attained, for all isotope-collimator combinations, if corrections for; scatter, attenuation, CDR (including septal scatter and penetration) and PVEs are performed. ^123^I LEHR and ^123^I ME quantification accuracies compared well when appropriate corrections for septal scatter and penetration were applied. This can be useful in departments that perform ^123^I studies and may not have access to ME collimators.

## Introduction

The branch theragnostics [1] has become a milestone in personalised cancer treatment, bridging the gap between Oncotherapy and Nuclear Medicine (NM). The principle of theragnostics lies in the combination of individualised targeted imaging of a cancer disease and its therapy. Both the diagnostic imaging and therapy procedures typically use the same pharmaceutical; however, in some cases, the diagnostic isotope may differ from that used in therapy. This allows for the visualisation of potential target volumes, such as tumours and organs at risk, enabling estimation of potential toxicities and predicting the benefits of such therapy [2, 3]. Metaiodobenzylguanidine (mIBG), a noradrenaline analogue, was developed in the early 1970s to image tumours in the adrenal medulla [4]. NM imaging using mIBG labelled to iodine-123 (^123^I) or iodine-131 (^131^I) has long employed the theragnostics concept. However, due to the recent development of peptide agents, the theragnostics approach has been heightened and the importance of accurate image quantification using ^123^I and ^131^I re-emphasised [2, 5, 6].

In the ^123^I decay, mainly photons are emitted (159.0 keV with 83% abundance, 27.3 keV with 71% abundance and 528.9 keV with 1% abundance) and the decay has a physical half-life of 13.2 h. ^131^I, with a physical half-life of 8.04 days, emits both photons (364.5 keV with 82% abundance, 636.9 keV with 7% abundance and 722.9 keV with 1.8% abundance) and several beta particles (with a maximum energy of 606.0 keV and mean energy of 192.0 keV). Due to its shorter half-life and absence of beta particles, ^123^I carries a reduced radiation burden compared to ^131^I, allowing for a higher administered activity amount for diagnostic imaging and is therefore better suited and favoured for diagnostic imaging. However, in centres where ^123^I is not available, ^131^I may be administered in lower activity quantities for diagnostic imaging [7, 8]. Both ^123^I-mIBG and ^131^I-mIBG accumulate in neuroendocrine tumours (NETs) as well as in the lungs, liver, kidneys, spleen, bladder, bone marrow and salivary glands [9, 10]. Therefore, it is essential that accurate activity uptake and related absorbed dose calculations are performed prior to therapy, to optimise the theragnostics’ effectiveness by quantitative imaging. Consequently, inaccuracies in the image quantification process may result in incorrect absorbed dose calculations and can lead to a reduction in therapeutic treatment efficacy. Physical factors which can potentially affect the accuracy in the activity estimation from an image are, the isotope-collimator combination used, the accuracy and precision in the scatter and attenuation correction methods, and the compensation for the limited spatial resolution that results in partial volume effects (PVEs).

High-energy (HE) collimators with thicker septa are needed when imaging ^131^I due to the relatively high primary photon energy of 364.5 keV, used for imaging, and because of the otherwise high septal scatter and penetration from the emission of the 636.9 keV and 722.9 keV photons [11]. However, ^123^I imaging can be performed with either a low-energy high resolution (LEHR) or a medium-energy (ME) collimator [12–14]. The ME collimator reduces the septal penetration and scatter from the higher-energy photons of ^123^I, resulting in improved image contrast and more accurate activity quantification. However, when appropriate septal scatter and penetration corrections are applied during image reconstruction, imaging with a LEHR collimator may very well be a useful alternative to a ME collimator [15, 16].

**Fig. 1:**
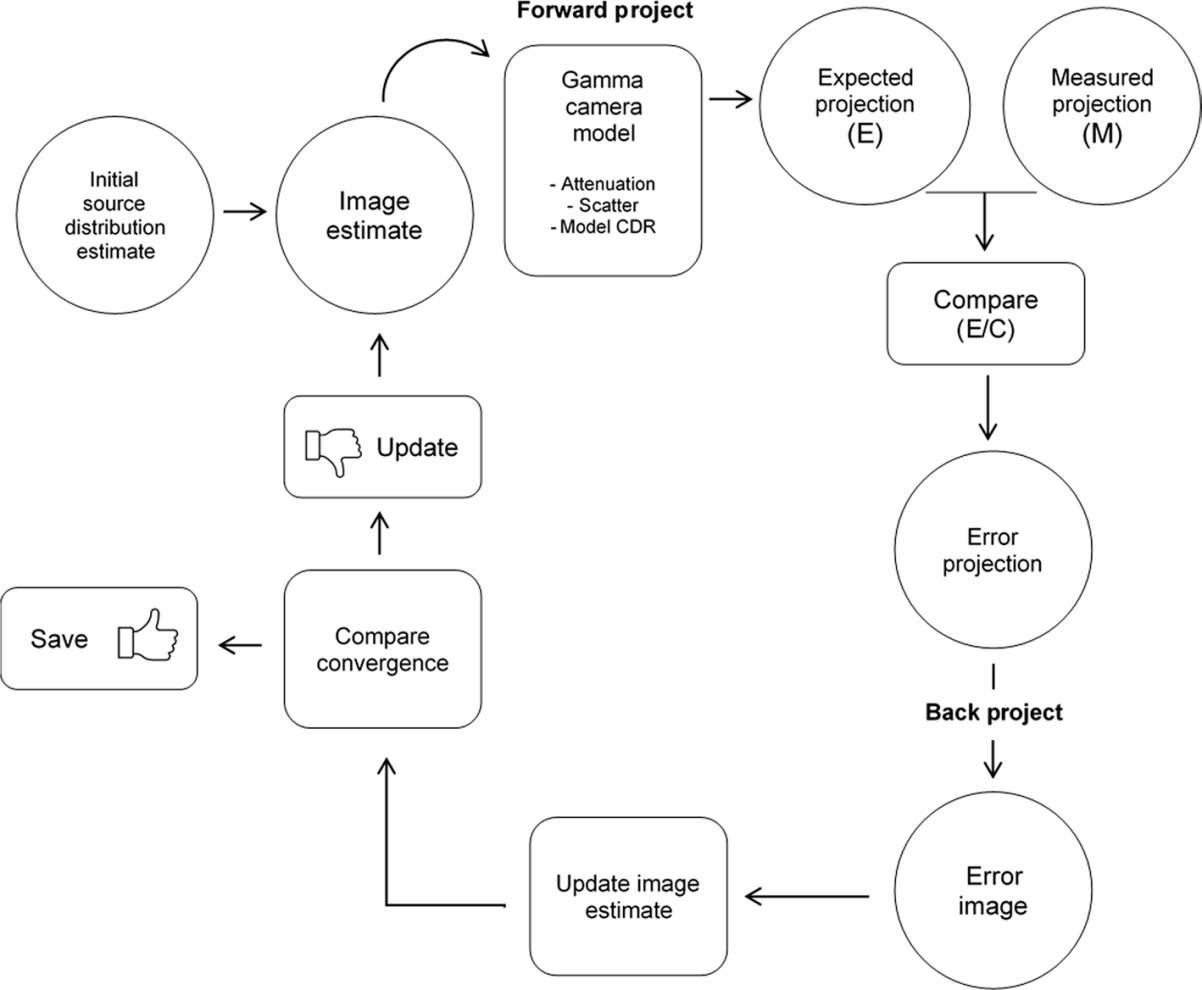
Schematic describing basic steps of iterative reconstruction method

Corrections for object scatter, non-homogenous attenuation and collimator detector response (CDR) as well as collimator-septal scatter and septal penetration, can be made by incorporating these effects in the camera/patient model, which is the essential part of any iterative reconstruction algorithm. The iterative maximum likelihood expectation maximisation (ML-EM) algorithm and the closely related ordered subset expectation maximisation (OS-EM) algorithm are routinely used and typically available on most commercial SPECT/CT systems [17, 18]. The basic steps in an iterative reconstruction algorithm are shown in Fig. 1. The main assumption is that if starting with an initial estimate of the activity distribution (blank or uniform image), the imaging process and the radiation transport are modelled in a forward projection to obtain the expected SPECT projections. These expected projections are then compared to measured projections, and an error projection is calculated as either the difference or the ratios of the expected and measured projections. The error projection is then back projected to create an error image. Once sufficient angles are processed (all or a subset of angles depending on the reconstruction algorithm), the error image is then used to update the source distribution estimate. The process is repeated (iterates) until the ratios converge to unity (i.e. maximising the likelihood). Because the expected and measured projections agree, it is assumed that the reconstructed images reflect the activity distribution in the patient and the process stops. However, the assumption is only valid if the same physical processes present in the real measurement are also modelled in the forward projection of the expected projection.

As the number of iterations increases, the reconstruction bias (difference between the estimated and measured data) is reduced, however, at the cost of image noise and reconstruction time. At a certain point, a further increase in the number of iterations will only increase image noise and reconstruction time [19]. Thus, it is important to determine the optimal number of OS-EM updates (number of subsets multiplied by the number of iterations) prior to reconstructing SPECT images for activity quantification purposes.

Another prerequisite for absolute SPECT activity quantification [20] is the conversion of reconstructed image counts to activity or activity concentration, using a gamma camera calibration factor (CF). Different methods of establishing such calibration factors (CFs) have been suggested. The simplest method is a planar acquisition of a small point-like source in air, where self-attenuation within the source is kept minimal, and corrections for scatter have been applied [21]. The gamma camera system sensitivity obtained from a Petri dish source in air, as defined by NEMA, has also been proposed [22]. However, according to the definition of system sensitivity, counts in the entire image are used. This may be problematic for isotope-collimator combinations such as ^123^I with the LEHR collimator and ^131^I with the HE collimator. For these isotopes-collimator combinations, a significant number of counts, originating from the higher energy photons which have penetrated the collimators’ septa, are detected within the main photopeak energy window. It is generally believed that a CF obtained with a source geometry that mimics the scatter and attenuation conditions in a patient is better suited [11]. SPECT acquisitions of geometries such as spheres in air or in water-filled cylindrical phantoms have been recommended, thereby reducing the effects of inadequate compensation for scatter and attenuation [20]. Both planar-based and SPECT-based CF methods rely on accurate corrections for scatter and attenuation [23, 24]. Unlike for planar imaging, corrections for scatter, attenuation and CDR are now often implemented in clinical workstations and thus can be routinely performed on SPECT/CT image data sets, making the SPECT-based CF the method of choice. Considering that the same corrections are applied to both the clinical and the CF data set, any effects of imperfect correction methods would be minimised.

In SPECT imaging, the partial volume effect (PVE) is the result of the gamma camera's finite spatial resolution and the discretisation of the measured coordinates from the gamma camera to a digital image and results in a reduction in image contrast between the regional uptake and surrounding areas. It is also a primary source-of-error when estimating activity in volumes-of-interest (VOIs) with sizes that are in the order of the camera’s resolution. Typically, VOIs smaller than three times the systems spatial resolution appear to contain less than actual activity concentrations [25]. PVEs are only partially compensated for when SPECT images are reconstructed with CDR compensations. Therefore, a simple and effective method for the correction of residual PVEs is to use recovery coefficients (RCs) determined for objects of similar sizes as the VOIs. RCs are often expressed as the ratio of the estimated activity concentration in a volume of interest (VOI) to the true activity concentration. RCs can be obtained from phantom measurements where the geometry of the sources and its activity concentrations are known or through Monte Carlo (MC) simulations, where RCs for more irregular and arbitrary shapes can be defined [26]. A curve of RCs as a function of object size can be generated and a recovery coefficient (RC) value for a relevant VOI size can then be applied in the activity quantification process [24, 27–29]. Often spherical sources are used to obtain RCs, but it should be noted that the RC also depends on the shape of the object [30]. Since the variation of tumours can vary significantly, it is hard to obtain RC curves for every possible tumour shape. Instead, it is common to compensate for PVEs of non-spherical tumours by applying volume-specific RCs, which were determined from spherical phantoms, but, this method has been reported to have limitations [31].

The MC method is an important tool to evaluate the quantification accuracy and its effects on the accuracy of dosimetry, for a given SPECT/CT procedure. The MC modelling of gamma cameras for imaging of isotopes, including technetium-99 m (^99m^Tc), ^131^I and lutetium-177 (^177^Lu), has been reported extensively [11, 32, 33]. There are various MC simulation codes available for NM imaging [34–36]. In this study we used the SIMIND MC code which models a standard clinical scintillation camera and has been successfully used to evaluate activity quantification accuracy [27, 37–39]. SIMIND allows for three methods to simulate the energy resolution, based on either (1) a simplified theoretical model assuming a $1/\sqrt{E}$ dependence [25, 40], (2) a fixed energy resolution used over the entire energy range or (3) a fitted model estimated from measured data [41]. A fitted energy resolution model improves the energy resolution modelling of high-energy photon emitting isotopes (e.g. ^131^I) and isotopes with multiple photopeaks (e.g. ^123^I, ^131^I and ^177^Lu) and was therefore used in this study. SIMIND has been validated for simulating ^123^I and ^131^I images with the proposed fitted energy resolution model by Morphis et al. [41].

Although activity quantification accuracy with SIMIND has been reported in the literature [27, 42, 43], little work has been done using voxel-based digital phantoms for ^123^I and ^131^I. Furthermore, to our knowledge, evaluating activity quantification accuracy in a clinically realistic phantom, using SIMIND with the fitted energy resolution model, has not yet been reported in the literature.

The aim of the study was to evaluate SPECT activity quantification accuracy for ^123^I with both the LEHR and ME collimators and ^131^I with the HE collimator, using the SIMIND MC code with a fitted energy resolution model. The objectives included (1) the optimisation of the number of OS-EM updates for the SPECT iterative reconstruction algorithm, (2) determination of a CF, (3) determination of RC curves and (4) evaluation of the quantitative method in voxel-based digital simple and patient phantoms.

## Materials and methods

The Siemens Symbia T16 (Siemens Healthcare, Erlangen, Germany) dual-head SPECT/CT system with LEHR, ME and HE collimators, in use at Universitas Academic Hospital (Bloemfontein, South Africa), was modelled using version 6.2 of the SIMIND MC code [39]. The set-up of the virtual gamma camera (detector and collimators) and the simulation specifications was the same as that described and validated by Morphis et al. [41, 44].

Voxel-based digital phantoms were created from CT images of a water-filled cylindrical phantom (internal diameter: 20.3 cm, external diameter: 21.6 cm and length: 31.7 cm) [45], as well as from a randomly selected retrospective patient SPECT/CT data set from the Universitas Academic Hospital patient database. These voxel-based digital phantoms were segmented and created as described by Ramonaheng et al. [27, 46] and Morphis et al. [41, 44].

SPECT projections images were simulated for ^123^I for both the LEHR and ME collimators (further referred to as ^123^I LEHR and ^123^I ME, respectively) and ^131^I using the HE collimator (^131^I HE). As part of the simulation process, Poisson noise was added to the projection data. Energy windows of 15.0% were centred over the 159.0 keV and 364.0 keV photopeaks of ^123^I and ^131^I, respectively. The images were simulated according to a standard clinical imaging protocol (step and shoot mode, non-circular orbit of rotation, 60 projections in total, 40 s per projection, matrix size: 128 × 128, pixel size: 4.8 × 4.8 mm^2^). Simulations were performed with a high number of histories (> 1 billion) [43].

SPECT reconstruction was performed using software developed at Lund University, Sweden [47], which incorporates an OS-EM iterative reconstruction package, developed by Frey and Tsui [48]. The iterative reconstruction algorithm performs a CT-based attenuation correction, model-based scatter correction by the effective scatter source estimation algorithm (ESSE), and a CDR correction, which accounts for collimator septal penetration and scatter by using pre-calculated MC simulated kernels [49]. The SPECT images were reconstructed in a 128 × 128 × 128 matrix with a voxel size of 4.8 × 4.8 × 4.8 mm^3^. Reconstructed image count statistics (total, mean and standard deviations) were obtained using the public domain software, Amide [50].

### Optimisation of OS-EM updates

When determining the optimal number of OS-EM updates, it is essential to consider the trade-off between bias, image noise and reconstruction time. To determine the optimal number of OS-EM updates and evaluate activity recovery as a function of the number of OS-EM updates, four spheres, with volumes of 1.8, 14.1, 47.7 and 113.1 mL (diameters: 1.5, 3.0, 4.5 and 6.0 cm), were digitally added to a segmented image of the water-filled cylindrical phantom. Different sized spheres were chosen to ensure coverage of at least one to four times the planar spatial resolution of ^131^I when imaged with the HE collimator (spatial resolution of 1.3 cm).

SPECT projections were simulated, with simulation parameters as explained above, for ^123^I and ^131^I, using sphere activity concentration values of 0.7 MBq/mL and 1.1 MBq/mL, respectively. The concentration values were based on clinical ^123^I and ^131^I mIBG tumour data at approximately 24 h post-administration, assuming diagnostic injected activity of 370.0 MBq and 185.0 MBq for ^123^I and ^131^I, respectively. The SPECT projections were reconstructed using LundADose, as described above. A series of 17 reconstructions were performed for each isotope-collimator combination, with six subsets, as proposed in the literature [11, 19], and OS-EM updates varying from 18 to 168. Count statistics (expressed as counts per second per mL) were obtained for each reconstructed sphere, with spherical VOIs equal to the sphere's physical size. Each VOI value was normalised to the overall maximum VOI value [33].

Brambilla et al. [51] and Leong et al. [52] evaluated reconstruction noise by assessing the change in the reconstruction noise level within a uniform area of a single slice. However, Ramonaheng et al. [27] suggested assessing the change in reconstruction noise level within a target VOI, obtained using the object’s physical size. This provides a constant and reliable delineation method, which is the same as that used to assess count recovery. As proposed by Ramonaheng et al. [27], the reconstruction noise levels in each sphere were calculated using Eq. 1,

1 $${\text{SD}}_{\rm relative} = { }\frac{\hbox{SD}}{{\hbox{mean}}} \times 100$$

where ${\text{SD}}_{\rm relative}$ is the relative standard deviation and *SD* and *mean* are the standard deviation and mean counts per voxel of the reconstructed counts from the VOI of the sphere. It is important to note that ${\text{SD}}_{\rm relative}$ is only an estimation of reconstruction noise [53]. Furthermore, in scenarios where CDR correction is included in the iterative reconstruction, the effect of the Gibbs artefact increases with an increase in the number of iterations; thus, the ${\text{SD}}_{\rm relative}$ will include this variation.

The normalised recovered counts and ${\text{SD}}_{\rm relative}$ values per sphere size were plot as a function of the number of OS-EM updates. The optimal number of OS-EM updates was determined from the recovered counts based on the graphs for each isotope-collimator combination and used in the subsequent reconstructions.

The optimal number of OS-EM updates was confirmed in a clinically realistic voxel-based digital patient phantom, where the activity recovery in a 14.1-mL (diameter: 3.0 cm) sphere was evaluated at several of the OS-EM updates.

### Calibration factors

For each isotope-collimator combination, four different source geometries were used to determine the gamma camera SPECT CFs. The source geometries included an activity-filled cylindrical phantom (CF_cylinder_), a 113.1 mL (diameter: 6.0 cm) activity-filled sphere in the water-filled (cold) cylindrical phantom (CF_water_), a 113.1 mL activity-filled sphere in air (CF_air_) and a point-like source (volume < 1 mL) in air (CF_point_). Concentration values for the four different CF source geometries were the same as the concentration value used to determine the optimal number of OS-EM updates.

SPECT projection images were simulated with the phantoms placed at the centre of the gamma camera's field of view and reconstructed using the optimised reconstruction parameters. Count statistics were obtained for each reconstructed CF source geometry. Cylindrical or spherical VOIs were defined based on the CF source geometry (cylindrical for CF_cylinder_ and spherical for the remaining geometries). Multiple VOI sizes with diameters of (1) less than the physical size, (2) the physical size plus a 3.0-cm margin or (3) the physical detector size were defined. To determine the CF_cylinder_, all three VOI diameters were used and VOI diameter (2) and (3) were chosen for the remaining CFs. Due to the presence of Gibbs artefacts in the CF_water_ and CF_air_ images, and to the limited size of the point source for CF_point_, VOIs smaller than the sources physical size were not considered. The CFs were calculated using Eq. 2,

2 $${\text{CF}} = { }\frac{{{\text{Cnt}}}}{A \times t} \left( {\frac{{{\text{cps}}}}{{{\text{MBq}}}}} \right)$$

where ${\text{Cnt}}$ is the total image counts obtained in each VOI, $A$ is the simulated activity in the source and $t$ the simulated acquisition time. From this, an appropriate CF source geometry was determined for all isotope-collimator combinations.

Energy spectra were also obtained from the CF_point_ SPECT simulations, for each isotope-collimator combination, to determine any possible contributions of septal scatter and penetration to the CFs.

### Recovery coefficient curves

RCs were determined for the dual-head Siemens Symbia T16 SPECT/CT gamma camera. Eight spheres, with volumes ranging from 8.2 to 523.6 mL (diameters: 2.5 to 10.0 cm), were digitally added to the segmented images of the water-filled cylindrical phantom. SPECT projections were simulated, with sphere activity concentration values and simulation parameters as explained above. Simulations were performed without background activity in the cylindrical phantom, to avoid any biased results in the quantification accuracy. The SPECT projections were reconstructed using the optimised reconstruction parameters. Count statistics with VOIs defined by the physical sphere size were obtained for each sphere using the predetermined CF geometry and reported in units of MBq/mL. The recovered and true activity concentration ($\left[ A \right]_{{{\text{recovered}}}}$ and $\left[ A \right]_{{{\text{true}}}}$) values were used to calculate the RCs, according to Eq. 3,

3 $${\text{RC}} = { }\frac{{\left[ A \right]_{{{\text{recovered}}}} }}{{\left[ A \right]_{{{\text{true}}}} }}$$

The RCs for each isotope-collimator combination were plotted as a function of sphere diameter and fitted with a mono-exponential function, as shown in Eq. 4, proposed by Willowson et al*.* [24],

4 $${\text{RC}} = a - be^{ - cx}$$

where $x$ represents the sphere diameter (cm), and $a$, $b$ and $c$ are the curve fitting parameters.

### Quantification

#### Spheres in the cylindrical phantom

To evaluate the quantitative method, the error of the estimated activity in spheres (volumes ranging from 8.2 to 523.6 mL) in an activity-filled cylindrical phantom was determined. Activity concentration values were based on clinical ^123^I and ^131^I mIBG pharmacokinetic data observed at 24-h post-injection (sphere to background ratio of 100:1). The recovered activity concentration was calculated using the predetermined CF and corrected for PVE using RCs obtained from the RC curve, for each isotope-collimator combination. The quantification error was defined as the percentage difference between the recovered activity concentration ($\left[ A \right]_{{{\text{recovered}}}}$) and the true activity concentration ($\left[ A \right]_{{{\text{true}}}}$) defined in SIMIND, shown in Eq. 5,

5 $${\text{Quantification}}\,{\text{Error}}\,(\%) = \frac{{\left[ A \right]_{{{\text{recovered}}}} - \left[ A \right]_{{{\text{true}}}} }}{{\left[ A \right]_{{{\text{true}}}} }}{ } \times 100.$$

#### Voxel-based digital patient phantoms

**Fig. 2:**
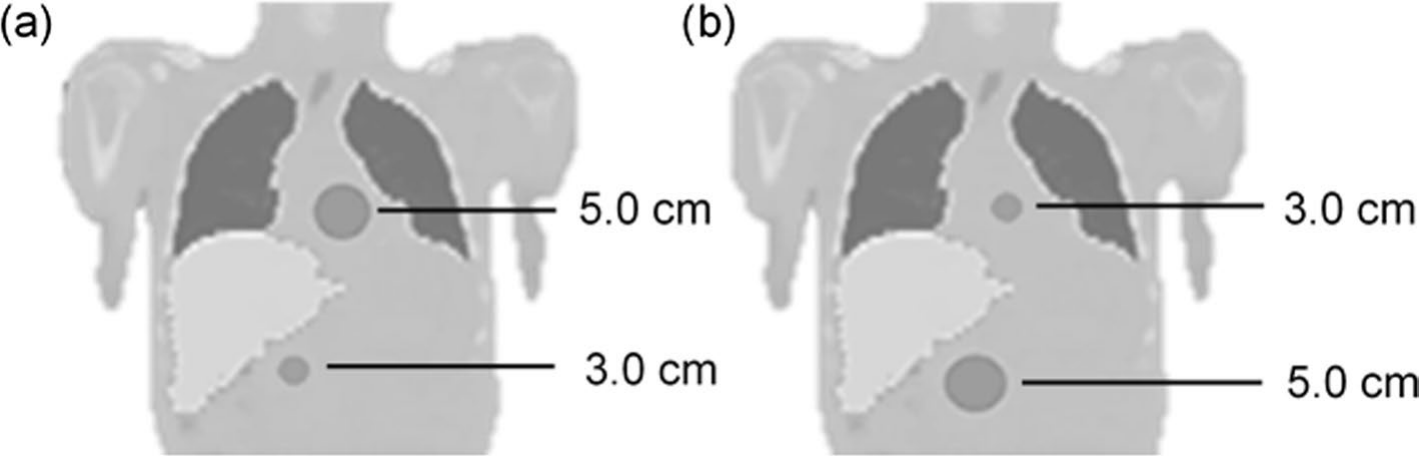
Segmented coronal CT slices of the voxel-based digital patient phantom with spheres' positions for **a** scenario 1 and **b** scenario 2

Two different sized spheres were added to a voxel-based digital patient phantom, to determine the accuracy in a clinically realistic phantom. As shown in Fig. 2, two scenarios were considered; scenario 1 included a sphere with a diameter of 5.0 cm positioned above the liver between the lungs, and a second sphere with a diameter of 3.0 cm positioned below the liver. For scenario 2, the positions of the two spheres were switched. Activity concentration values were based on clinical ^123^I and ^131^I mIBG pharmacokinetic data observed at 24-h post-injection (sphere to background ratio of 100:1, sphere to liver ratio of 100:7.5 and sphere to lung ratio of 100:2.8).

^123^I and ^131^I SPECT projections were simulated and reconstructed as previously described. Count statistics were obtained with VOIs defined according to the physical size of the spheres and the liver. Sphere and liver activity concentrations were calculated using the predetermined CF geometry and corrected for PVE using the appropriate RC obtained from the RC curve, and the error in the recovered activity concentration was determined using Eq. 5.

## Results

### Optimisation of OS-EM updates

**Fig. 3:**
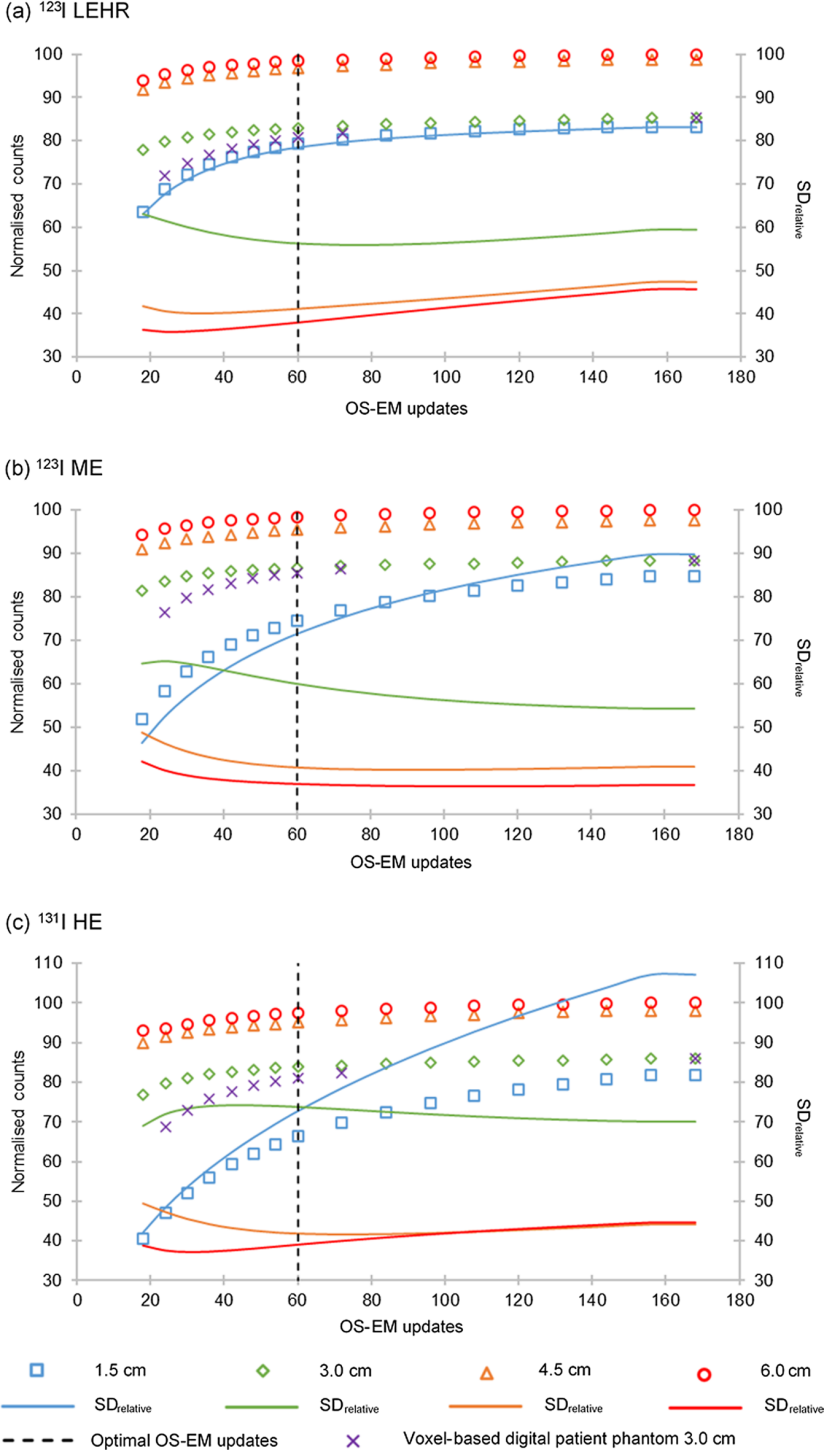
Normalised counts and ${\text{SD}}_{{{\text{relative}}}}$ as a function of OS-EM updates for spheres with diameters of 1.5 cm, 3.0 cm, 4.5 cm, and 6.0 cm, for **a** ^123^I LEHR, **b** ^123^I ME and **c** ^131^I HE

The relationship between the number of OS-EM updates and (1) the recovered counts and (2) ${\text{SD}}_{{{\text{relative}}}}$, for each sphere size is illustrated in Fig. 3 for all isotope-collimator combinations.

Figure 3a–c shows that count convergence (fast increase followed by a slower approach towards an asymptote) is reached relatively quickly for the three largest spheres, compared to the smallest sphere. Eighteen OS-EM updates resulted in more than 90% count recovery for the two larger spheres (4.5 cm and 6.0 cm), for all isotope-collimator combinations. However, more OS-EM updates were required to reach count convergence for the two smallest spheres (1.5 cm and 3.0 cm). This can be attributed to the limited spatial resolution and the PVE of ^123^I LEHR, ^123^I ME and ^131^I HE [16].

The graphs in Fig. 3 show that for all isotope-collimator combinations reaching 60 OS-EM updates, a further increase in OS-EM updates would not improve count recovery by more than 3.0% for the three larger spheres and 16.0% for the smallest sphere. The change in ${\text{SD}}_{{{\text{relative}}}}$ from 18 OS-EM updates to 60 OS-EM updates was less than 8.0% for the three larger spheres. The smallest sphere resulted in an ${\text{SD}}_{{{\text{relative}}}}$ increase of 15.0% (improved count recovery of 16.0%), 16.0% (improved count recovery of 23.0%) and 25.0% (improved count recovery of 26.0%) for ^123^I LEHR, ^123^I ME and ^131^I HE, respectively. The count recovery improved by a maximum of 1.0% for the three largest spheres (2.0% increase in ${\text{SD}}_{{{\text{relative}}}}$) and 6.0% for the smallest sphere (9.0% increase in ${\text{SD}}_{{{\text{relative}}}}$), when moving from 54 OS-EM updates to 72 OS-EM updates, across all isotope-collimator combinations. With the goal of accurate activity quantification, an improvement in count recovery was favoured. However, based on only a 1.0% increase in count recovery from 54 OS-EM updates to 72 OS-EM updates (excluding the smallest sphere), 60 OS-EM updates were considered optimal for all isotope-collimator combinations.

This was confirmed by the recovered activity in the 3.0 cm sphere (mimicking a tumour) in the voxel-based digital patient phantom. The average increase in count recovery from 54 OS-EM updates to 72 OS-EM updates was 1.8%. A further increase in the number of OS-EM updates did not improve count recovery by more than 4.7%.

If the goal is to perform accurate activity quantification for small objects (1.5 cm and less), it may be considered to further increase the number of OS-EM updates, keeping in mind the simultaneous increase in image noise. It should also be noted that for the smallest sphere, full recovery is not reached at 168 OS-EM updates; however, the use of a RC could further improve the quantification accuracy.

### Calibration factors

Table 1 shows the CFs for the different source geometries and VOI diameters. The relative SDs for the average of the four CFs, when selecting a VOI diameter equal to the physical source diameter plus 3.0 cm, were 2.9%, 1.1% and 1.5% for ^123^I LEHR, ^123^I ME and ^131^I HE, respectively. Similar results were obtained when selecting the VOI equal to the detector’s physical size (relative SDs of 1.9%, 0.8% and 4.7% for ^123^I LEHR, ^123^I ME and ^131^I HE, respectively). For ^123^I LEHR, the selection of the different VOI margins resulted in a 5.0% difference in CF between VOI selection (1) and (2), 13.0% difference in CF between VOI selection (1) and (3) and 19.0% difference in CF between VOI selection (2) and (3). The corresponding differences obtained for ^123^I ME were all less than 2.2%. ^131^I HE resulted in differences of 1.1%, 7.2% and 8.4%, respectively. These results show that the effect of VOI selection is more pronounced for ^123^I LEHR and ^131^I HE. This can be attributed to unwanted septal scatter and penetration, which was not fully corrected for using the OS-EM reconstruction and the appropriate correction methodologies.

**Table 1:** CFs for ^123^I with the LEHR and ME collimator and ^131^I with the HE collimator, in cps/MBq, for the four CF source geometries

VOI diameter		^123^I LEHR (cps/MBq)	^123^I ME (cps/MBq)	^131^I HE (cps/MBq)
1	CF_cylinder_	89.3	119.0	27.7
2	CF_cylinder_	88.4	115.6	26.8
	CF_water_	84.4	118.2	27.6
	CF_air_	82.4	116.4	27.2
	CF_point_	84.1	115.5	27.8
	Average ± SD	84.8 ± 2.5	116.4 ± 1.3	27.4 ± 0.4
	Relative SD (%)	2.9	1.1	1.5
3	CF_cylinder_	100.4	118.9	28.6
	CF_water_	102.2	120.2	29.1
	CF_air_	98.4	117.7	29.4
	CF_point_	102.4	118.7	31.7
	Average ± SD	100.9 ± 1.9	118.9 ± 1.0	29.7 ± 1.4
	Relative SD (%)	1.9	0.8	4.7

VOI diameters were defined as (1) less than the physical source diameter, (2) equal to the physical source diameter plus a 3-cm margin and (3) equal to the physical size of the detector

**Fig. 4:**
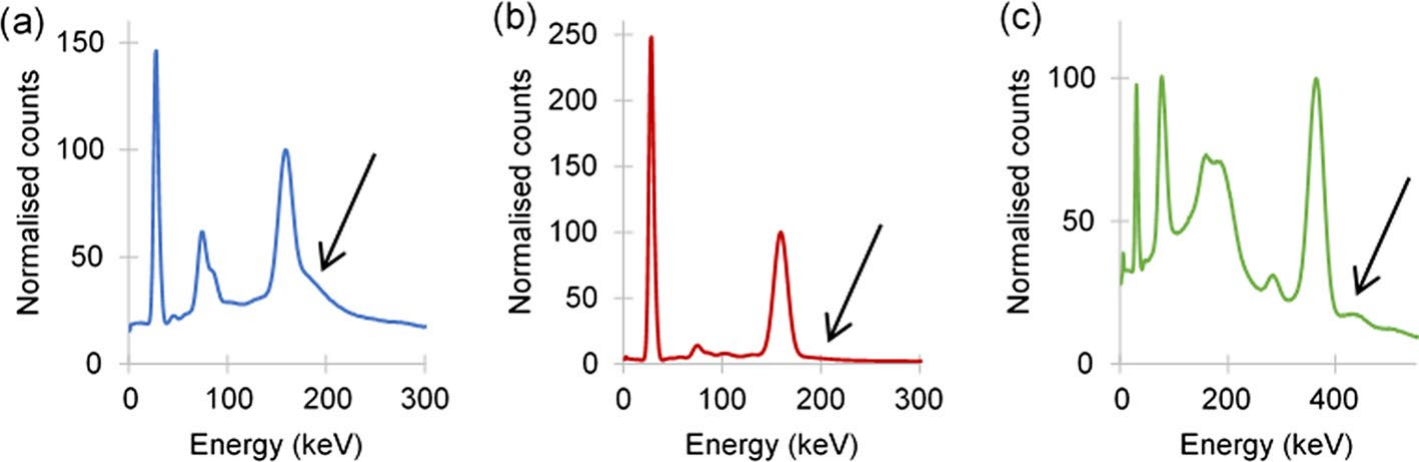
Simulated energy spectra of CF_point_ projection images for **a** ^123^I LEHR, **b** ^123^I ME and **c** ^131^I HE

To better visualise the effect of septal scatter and penetration, simulated energy spectra of CF_point_ projection images are shown in Fig. 4. Higher levels of septal penetration (indicated by the arrows) can be seen in Fig. 4a compared to Fig. 4b, c. This further reiterates the importance of accurate septal scatter and penetration corrections, which could be the cause of the larger difference between CFs with VOI diameters (1) and (2) to that of (3) for ^123^I LEHR, as shown in Table 1. For this reason, the use of VOI diameter (3) was not considered further.

When considering the use of I-131 in the clinic, considering the high-cost implications, the possibility of contamination, the high radiation risk to staff members due to its high photon energy, emission of beta particles, long half-life and volatility, as well as its impracticality of use in the clinical environment, the use of CF_cylinder_ was not considered further, despite being recommended by others [29, 62]. For this reason, VOI diameter (1) was also not considered further. When comparing the results of the remaining three geometries under the remaining VOI diameter (2), maximum differences of 2.0 cps/MBq (between CF_water_ and CF_air_), 3.7 cps/MBq (between CF_water_ and CF_point_) and 0.6 cps/MBq (between CF_air_ and CF_point_) were noted for ^123^I LEHR, ^123^I ME and ^131^I HE, respectively.

Based on these findings, the CF_point_ with a VOI diameter equal to the physical size plus a 3.0-cm margin, representing the simplest geometry and easiest to replicate in the clinical environment, was considered appropriate to use as a CF for all isotope-collimator combinations.

### Recovery coefficient curves

**Fig. 5:**
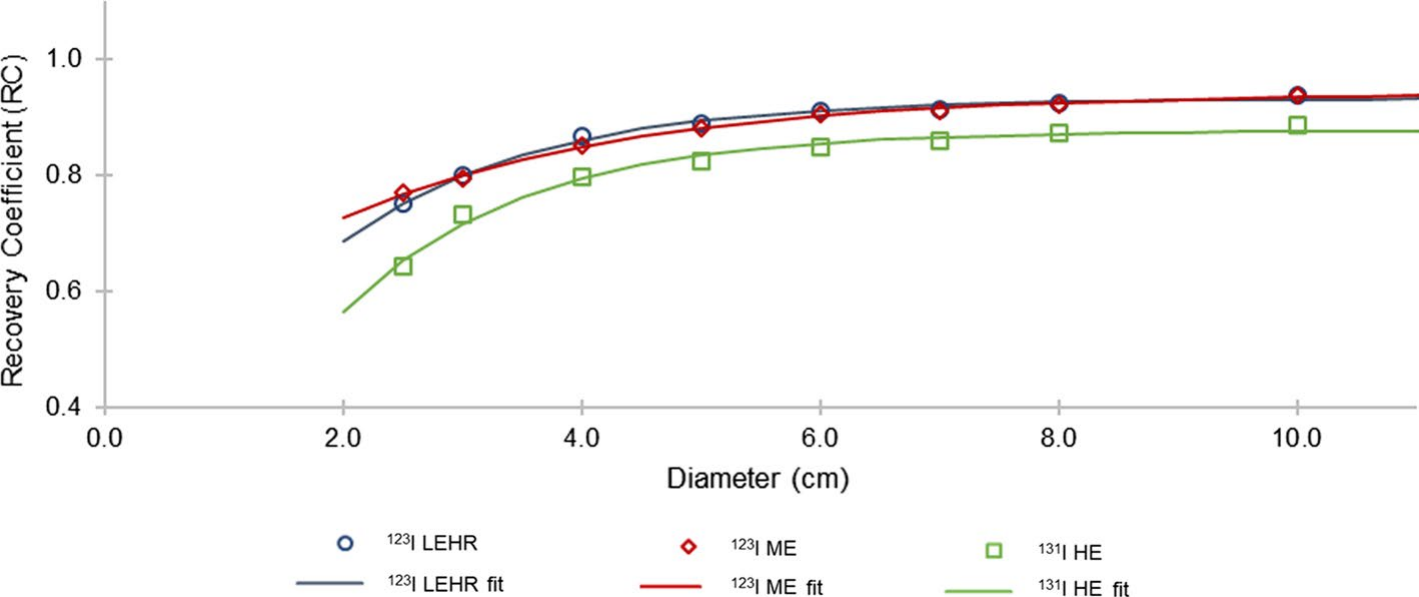
RC as a function of sphere diameter for ^123^I LEHR, ^123^I ME and ^131^I HE, with the solid lines showing the fitted functions

Figure 5 shows the calculated RCs as a function of sphere diameter for all isotope-collimator combinations. The mono-exponential functions fitted to the data, allowing for interpolation between sphere sizes, had R^2^ values of 0.99 for all isotope-collimator combinations. From Fig. 5 it is evident that for ^123^I LEHR and ^123^I ME, more than 90.0% (RC > 0.9) of the activity concentration is recovered for spherical sources with diameters equal to and larger than 5.0 cm. Comparatively, more than 80.0% (RC > 0.8) of the activity concentration was recovered for ^131^I HE at sphere diameters of 5.0 cm and more, and 90.0% (RC > 0.9) at sphere diameters equal to and larger than 7.0 cm. This emphasises the impact of PVEs for isotope-collimator combinations with poorer spatial resolution, such as ^131^I HE.

### Quantification

#### Spheres in the cylindrical phantom

**Fig. 6:**
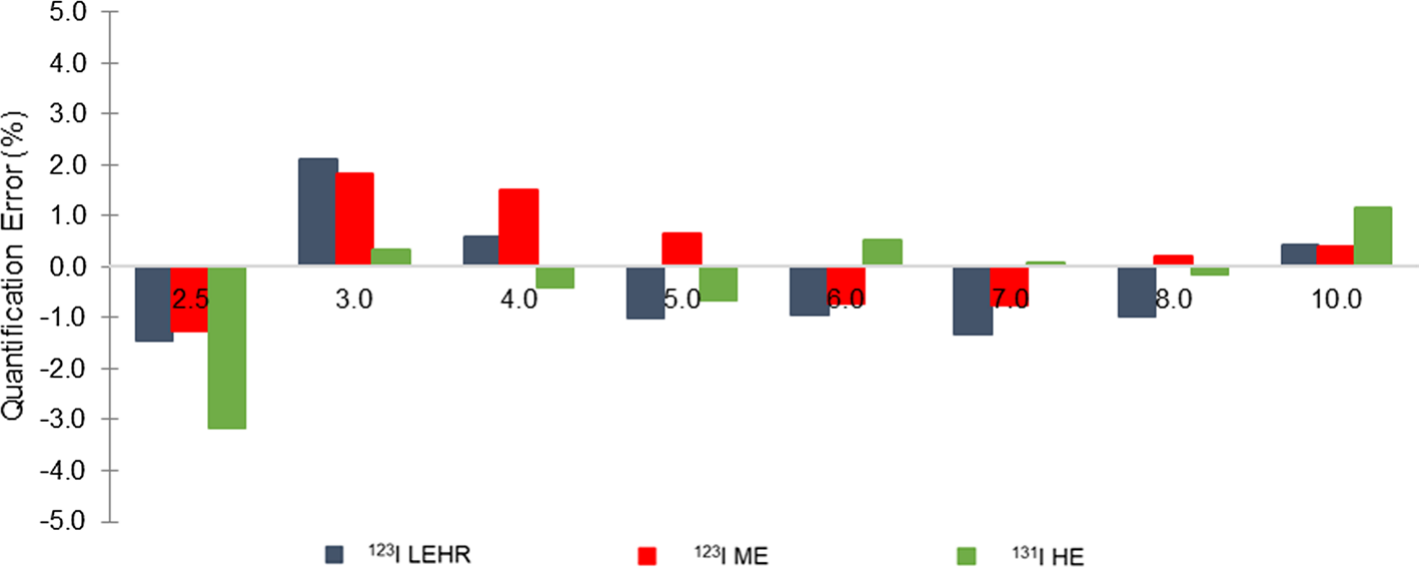
Quantification error (%) of spheres of differing diameter in a warm cylindrical phantom, for ^123^I LEHR, ^123^I ME and ^131^I HE

The quantification error, as defined by Eq. 5, for spheres with diameters ranging from 2.5 to 10.0 cm, in a warm cylindrical phantom, obtained with the CF and recovery curves, as determined in Sects. 2.2 and 2.3, respectively, for all isotope-collimator combinations, is shown in Fig. 6. Overall, percentage quantification errors were less than 3.2% for all isotope-collimator combinations.

#### Voxel-based digital patient phantoms

**Fig. 7:**
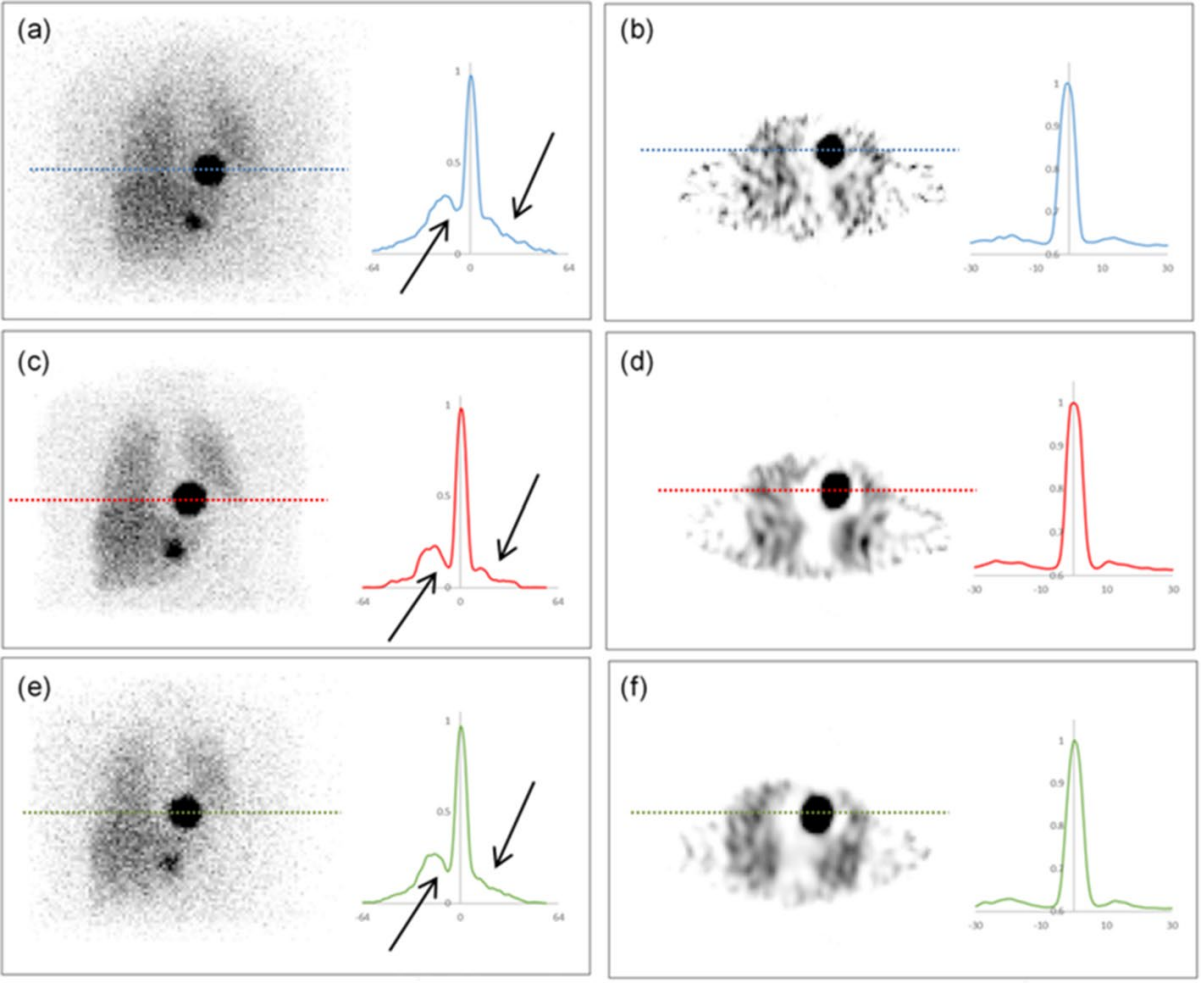
Uncorrected projection images (a, c, e), and reconstructed corrected transaxial slices (b, d, f) through the voxel-based digital patient phantom for **a**, **b** ^123^I LEHR, **c**, **d** ^123^I ME and **e**, **f** ^131^I HE. Profiles through the largest sphere are displayed alongside their respective images. Arrows indicate septal scatter and penetration at peak and trough and at tail region

Figure 7 shows projection images and reconstructed transaxial slices of the voxel-based digital patient phantom for all isotope-collimator combinations. Additionally, profiles through the largest sphere (diameter: 5.0 cm) are shown alongside their respective images.

**Fig. 8:**
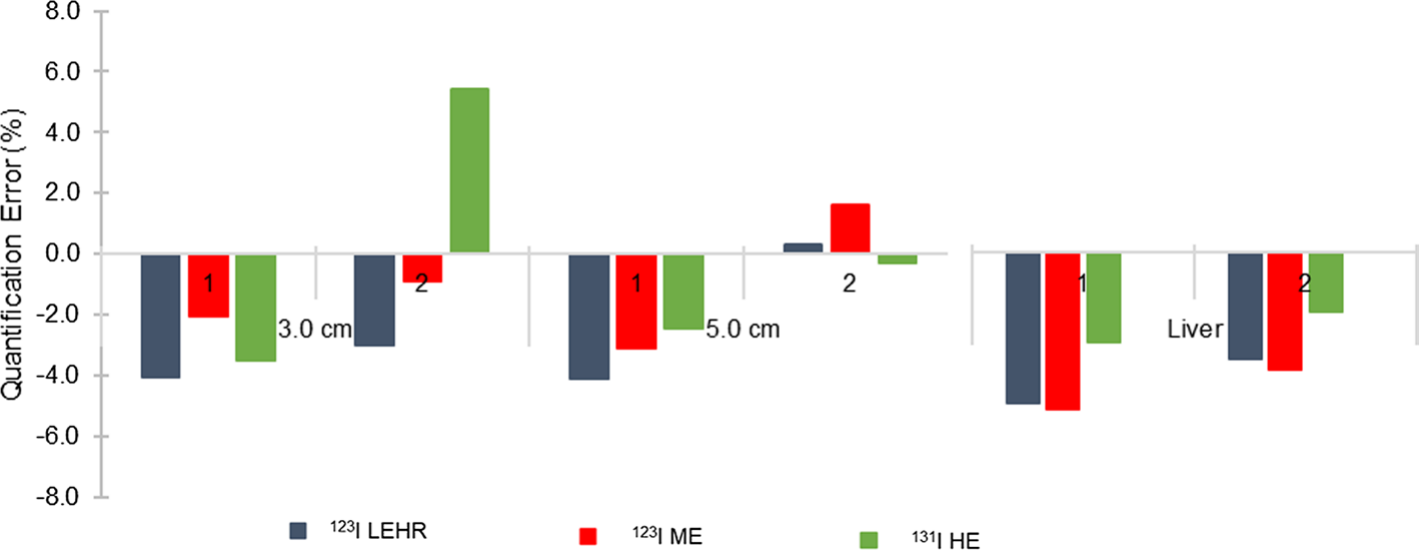
Activity quantification errors (%) for spheres in the voxel-based digital patient phantom (scenario 1 and 2), for ^123^I LEHR, ^123^I ME and ^131^I HE

Differences in background counts due to collimator septal scatter and penetration can be seen between ^123^I LEHR and ^123^I ME in Fig. 7a, c, respectively. The presence of septal scatter and penetration is further illustrated with the profile through the 5.0 cm sphere, as shown by the arrows in Fig. 7a compared to Fig. 7c. However, comparing the reconstructed slices and the profile through the 5.0 cm sphere, as shown in Fig. 7b, d, it can be seen that septal scatter and penetration is sufficiently compensated for the case of ^123^I LEHR (ratio of peak to trough counts increased). Overall, the ^123^I LEHR images appear slightly noisier compared to ^123^I ME. This can be attributed to poorer counts statistics due to the reduced sensitivity of ^123^I LEHR compared to ^123^I ME, considering equal amounts of activity were simulated in each. The effects of septal scatter and penetration are notably lower for ^131^I HE, as shown in Fig. 7e, f. Visually, the ^123^I ME images are comparable to the ^131^I HE images. Similar differences have been reported in the literature by Nakajima et al. [54].

The activity quantification errors in the two spheres are shown in Fig. 8 for all isotope-collimator combinations. The 3.0 cm and 5.0 cm spheres show an absolute quantification error of less than 5.4% for all isotope-collimator combinations for both scenarios 1 and 2. For the liver, absolute quantification errors of less than 5.2% are noted across all isotope-collimator combinations.

## Discussion

The purpose of our work was to evaluate the accuracy of activity quantification for SPECT imaging when using ^*123*^*I* with both the LEHR and ME collimators, and for ^*131*^*I* with the HE collimator.

## Optimisation of OS-EM updates

From the results, it is evident that optimisation of the number of OS-EM updates is important for accurate SPECT activity quantification. The literature states that smaller objects require a larger number of OS-EM updates to improve reconstruction accuracy [19], which corroborates our results, as shown in Fig. 3.

It is well known that increasing the number of OS-EM, increases image noise. The literature supports this statement for several isotopes, including ^99m^Tc [52], ^131^I [43, 55, 56] and ^177^Lu [27, 57, 58]. The results in Fig. 3 corroborate that stated in literature, showing that the image of the smallest sphere has the largest ${\text{SD}}_{{{\text{relative}}}}$ value and that the value decreases as the sphere size increases.

Figure 3 also shows that the optimal number of OS-EM updates is object size-dependent. Based on these findings, the optimal number of OS-EM updates was found to be 60 for all isotope-collimator combinations. Our study's findings were similar to results reported in the literature [11, 19, 59]. The choice of optimal number of OS-EM updates was confirmed by similar convergence obtained with the voxel-based digital patient phantom (Fig. 3).

## Calibration factors

The literature reports the use of several CFs, which include the activity-filled cylindrical phantom, point-like or spherical source in air [20, 21]. The simplest way of determining a CF is from a point-like source in air. However, this method relies on SPECT data which has been reconstructed with high accuracy. To account for any uncertainties in the reconstruction process, a more reliable source geometry is one which better approximates the patients’ scatter and attenuation properties [43]. However, Dewaraja et al. [43] also stated that when comparing ^131^I HE quantification results obtained with a spherical (volume: 200.0 ml, diameter: 7.3 cm) and a point source CF, there was no evidence that one geometry outperformed the other.

As with D'Arienzo et al. [20], the relative percentage SD values displayed in Table 1 show little difference between the four CF source geometries (CF_cylinder_, CF_water_, CF_air_ and CF_point_). However, this small error may not always be representative, as the error is highly dependent on the compensation methods implemented in the clinical reconstruction algorithm. The reconstruction algorithm used in this study includes compensation for object scatter and attenuation as well as CDR which, in turn, compensates partly for collimator septal scatter and penetration. This is not always true for software implemented on clinical workstations.

Our results showed the importance of the choice of VOI size when obtaining a CF. This is especially important when imaging isotope-collimator combinations resulting in high levels of septal scatter and penetration, as seen in Fig. 4a, c for ^123^I LEHR and ^131^I HE, respectively. Despite the reconstruction process accounting for septal scatter and penetration, counts from septal scatter and penetration counts are still present, as evident in the results shown in Table 1. The difference in CF when using VOI diameters (2) and (3) is 18.9% and 8.6% for ^123^I LEHR and ^131^I HE, respectively. However, due to the minimal amount of septal scatter and penetration present with ^123^I with the ME collimator, the difference in CF values between VOI diameters (2) and (3) is less than 2.2%.

The results also show that a less demanding CF source geometry can be used, if appropriate corrections for scatter, attenuation and CDR (including septal scatter and penetration) are applied during reconstruction. For this reason, the CF_point_ was considered optimal for all isotope-collimator combinations, if a VOI diameter equal to the physical size, extended with a radial distance of 3.0 cm as a margin, is used.

## Recovery coefficient curves

According to the literature, the shape of the recovery curves is dependent on several factors, including the size and shape of the object, as well as the presence of activity surrounding the object. [60]. In this work, only spherical sources, placed in a water-filled cylindrical phantom, were used to determine the RC curves (Fig. 5) for the three isotope-collimator combinations. As a consequence, when applying these RCs as PVE corrections for structures with irregular shapes, the result may be inaccurate [31]. It is important to note that the CF source geometry chosen will influence the RCs [27]. In this study, the CF was obtained from the CF_point_ with a VOI diameter equal to the physical size plus a 3.0-cm margin. However, the VOI diameter used to determine the recovered activity concentration for the RCs was set equal to the sphere's physical size, since this VOI definition was used to obtain the activity quantification accuracy in the voxel-based digital phantoms. This explains why the RC curves do not reach a value of 1, for all isotope-collimator combinations. The underestimation of the true activity concentration was more pronounced for ^131^I HE due to the collimator's limited spatial resolution [60]. Similar results have been reported in the literature for ^131^I, where different VOI geometries were used to obtain RCs and CFs [61].

## Quantification

Figure 6 shows absolute quantification errors not more than 3.2% for spheres in an activity-filled cylindrical phantom. The position dependence of VOIs is more pronounced for smaller objects (especially those smaller than two to three times the spatial resolution), which typically results in poorer quantification accuracies. It is important to keep in mind that due to PVEs, smaller objects are generally associated with larger quantification errors [61].

Dewaraja et al. [19] summarised the results from physical phantom evaluations of SPECT quantification and highlighted that for ^131^I activity quantification without compensation for PVEs, errors of 17.0% for large spheres (8.0 mL to 95.0 mL), and up to 31.0% for small spheres (4.0 mL) [55] were obtained. In another study by Koral et al. [62], ^131^I quantification errors of less than 7.0% were reported when compensation for PVE was applied. These results highlight the importance of using appropriate PVE corrections to obtain accurate activity quantification. For ^123^I LEHR activity quantification, errors of up to 5.0% were reported by Shcherbinin et al. [23].

In a recent study, Westerberg [61] reported ^131^I activity quantification errors up to 13.0% and 8.0% for small (10.0 mL) and large (26.0 mL) spheres, respectively. The author reported an activity quantification error of 35.0% for the kidney, when no compensation for PVE was applied. Brady et al. [12] reported ^123^I activity quantification errors of 10.0% for spheres with volumes ranging between 5.8 mL and 29.0 mL.

Our study results showed smaller quantification errors in comparison with results in some of the literature findings, which is most likely attributed to differences in the compensation and reconstruction methods used and the addition of PVE compensation which was often omitted by the referenced authors.

The bias difference noted for the 3.0 cm sphere in patient scenario 2, for ^131^I HE is striking; however, results are only shown for a single patient. We therefore speculate that this may be due to statistical variations; however, it is recommended that future work include more patient phantoms.

## Conclusion

Our results show when using a simple CF source geometry in clinically realistic patient geometries, it is possible to obtain activity quantification accuracies within 6.0% for all isotope-collimator combinations, if appropriate corrections for scatter, attenuation, CDR and PVEs were incorporated into the reconstruction of the SPECT data. Our results also show that similar ^123^I activity quantification accuracies could be obtained with the LEHR and ME collimators.

The quantification procedure suggested in this study can easily be implemented in routine clinical practice. I-123 imaging should preferably be performed with a ME collimator. However, if appropriate corrections for septal scatter and penetration can be applied, then the ^123^I LEHR combination can be useful for departments that do not have access to a ME collimator.

## Data Availability

The data sets generated during the study are available from the corresponding author on reasonable request.

## Abbreviations

^99m^Tc: Technetium-99 m
^123^I: Iodine-123
^131^I: Iodine-131
^177^Lu: Lutetium-177
CDR: Collimator detector response
CF: Calibration factor
CFs: Calibration factors
HE: High energy
LEHR: Low-energy high resolution
MC: Monte Carlo
ME: Medium energy
mIBG: Metaiodobenzylguanidine
ML-EM: Maximum likelihood expectation maximisation
NETs: Neuroendocrine tumours
NM: Nuclear medicine
OS-EM: Ordered subset expectation maximisation
PVE: Partial volume effect
PVEs: Partial volume effects
RC: Recovery coefficient
RCs: Recovery coefficients
VOI: Volume of interest
VOIs: Volumes of interest

## Code availability

SIMIND is freely available for download (www.msf.lu.se/research/simind-monte-carlo-program/downloads).
